# Exploring the Impact of Hepatic Impairment on Pralsetinib Pharmacokinetics

**DOI:** 10.3390/pharmaceutics16040564

**Published:** 2024-04-20

**Authors:** Kit Wun Kathy Cheung, Yang Tang, Doreen Anders, Teresa Barata, Astrid Scalori, Priya Agarwal, Rucha Sane, Sravanthi Cheeti

**Affiliations:** 1 Clinical Pharmacology, Genentech, Inc., South San Francisco, CA 94080, USA; 2 Drug Metabolism and Pharmacokinetics, Genentech, Inc., South San Francisco, CA 94080, USA; 3 Clinical Safety, F. Hoffmann-La Roche Ltd., 4058 Basel, Switzerland; 4 Data and Statistical Science, F. Hoffmann-La Roche Ltd., 4058 Basel, Switzerland; 5 Clinical Development Oncology, F. Hoffmann-La Roche Ltd., Welwyn Garden City AL7 1TW, UK

**Keywords:** hepatic impairment, pralsetinib, pharmacokinetics

## Abstract

Pralsetinib is a kinase inhibitor indicated for the treatment of metastatic rearranged during transfection (*RET*) fusion-positive non-small cell lung cancer. Pralsetinib is primarily eliminated by the liver and hence hepatic impairment (HI) is likely alter its pharmacokinetics (PK). Mild HI has been shown to have minimal impact on the PK of pralsetinib. This hepatic impairment study aimed to determine the pralsetinib PK, safety and tolerability in subjects with moderate and severe HI, as defined by the Child–Pugh and National Cancer Institute Organ Dysfunction Working Group (NCI-ODWG) classification systems, in comparison to subjects with normal hepatic function. Based on the Child–Pugh classification, subjects with moderate and severe HI had similar systemic exposure (area under the plasma concentration time curve from time 0 to infinity [AUC_0–∞_]) to pralsetinib, with AUC_0–∞_ geometric mean ratios (GMR) of 1.12 and 0.858, respectively, compared to subjects with normal hepatic function. Results based on the NCI-ODWG classification criteria were comparable; the AUC_0–∞_ GMR were 1.22 and 0.858, respectively, for subjects with moderate and severe HI per NCI-ODWG versus those with normal hepatic function. These results suggested that moderate and severe hepatic impairment did not have a meaningful impact on the exposure to pralsetinib, thus not warranting a dose adjustment in this population.

## 1. Introduction

Pralsetinib is a potent and selective oral inhibitor of rearranged during transfection (*RET*) fusion proteins and oncogenic *RET* mutants [1]. It is approved in the United States for treatment of adult patients with metastatic *RET* fusion-positive non-small cell lung cancer (NSCLC) and adults and pediatric patients 12 years and older with advanced or metastatic *RET* fusion-positive thyroid cancer who require systemic therapy and who are radioactive iodine-refractory [2,3].

Pharmacokinetic (PK) analysis was performed following single oral doses of pralsetinib ranging from 60 to 600 mg in RET fusion-positive NSCLC patients [2,3]. Pralsetinib was rapidly absorbed, with a median time to maximum observed concentration (t_max_) around 2 to 4 h postdose. The mean plasma elimination half-life (t_1/2_) was 15.7 h following a single oral dose of 400 mg and 20 h following multiple once-daily 400 mg oral dosing of pralsetinib. The oral bioavailability of a 200 mg dose of pralsetinib increased when given under fed conditions. Moreover, food significantly delayed the absorption of pralsetinib when compared to fasted conditions. Therefore, the recommended dosage of pralsetinib in adults and adolescents is 400 mg administered orally once daily (QD) on an empty stomach.

In vitro studies and clinical drug–drug interaction (DDI) studies suggested that pralsetinib is a substrate of P-glycoprotein (P-gp) and cytochrome P450 3A4 (CYP3A4) enzymes [4]. In DDI studies with itraconazole (a CYP3A and P-gp inhibitor) and cyclosporine (P-gp inhibitor), pralsetinib exposure increased by 3.5-fold and 1.8-fold, respectively [5]. In addition, a DDI study with rifampin, a strong CYP3A inducer, showed a 68% reduction in pralsetinib exposure [2,3].

Results from a human mass balance study demonstrated that pralsetinib is eliminated primarily via feces, as 73% of the radioactive dose was recovered in feces and 6% in urine [4]. Hepatic impairment can alter drug exposures for drugs that are primarily eliminated in the liver. Population PK (popPK) analysis of pralsetinib suggested that mild hepatic impairment did not impact the pralsetinib PK in patients, based on which it was concluded that no dose adjustment is required for these patients [2,3,6]. However, a dedicated clinical study to understand the impact of moderate and severe hepatic impairment on the pralsetinib PK was warranted to determine if there is a need to adjust pralsetinib dose for these patients.

This study aimed to evaluate the effects of moderate and severe hepatic impairment on the PK and safety profile of pralsetinib compared to healthy subjects, thereby assisting in providing dosing recommendations for pralsetinib in these patients based on the Child–Pugh classification [7,8,9]. These patients were re-classified per the National Cancer Institute Organ Dysfunction Working Group (NCI-ODWG) for an exploratory analysis as most oncologists use this classification system to evaluate the hepatic function of their patients [10].

## 2. Materials and Methods

### 2.1. Study Design and Treatment

This study was an open-label, multi-center, single-dose, parallel-group, safety, tolerability, and PK study of pralsetinib administered at 200 mg to fasted males and females of non-childbearing potential with varying degrees of hepatic impairment.

**Figure 1:**
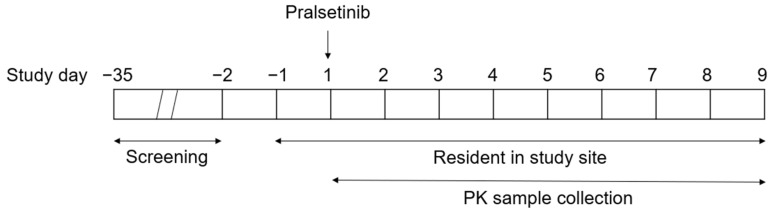
Study Schema.

The study schema is depicted in Figure 1. Enrolled subjects were admitted to the study site on the day prior to dosing (Check-in on Day −1) to collect their baseline data. Subjects were confined at the study site from the time of Check-in until Clinic Discharge on Day 9 (study completion). Subjects received a single 200 mg pralsetinib oral dose after an overnight fast of at least 8 h from food (not including water), followed by fasting from food for at least 2 h post dose.

This study included subjects with moderate or severe hepatic impairment and matching subjects with normal hepatic function. Up to 32 subjects were allowed to enroll to complete the study with a minimum of 18 subjects, with at least 6 subjects in each of the following cohorts: Cohort 1 (enrolled up to 16 subjects with normal hepatic function); Cohort 2 (enrolled up to 8 subjects with moderate hepatic impairment with Child–Pugh score 7 to 9, inclusive, who showed decreased albumin and/or elevated bilirubin levels); or Cohort 3 (enrolled up to 8 subjects with severe hepatic impairment with Child–Pugh score 10 to 15, inclusive, who showed decreased albumin and/or elevated bilirubin levels). No formal statistical sample size estimation was performed; this sample size was determined based on the regulatory guidance [7,9]. Subjects with normal hepatic function were dosed as a healthy control group, matched 1:1 to enrolled subjects with hepatic impairment with respect to age (±5 years), body weight (±10%), and sex. To reduce the number of subjects enrolled, subjects with normal hepatic function were matched to more than 1 subject with hepatic impairment across the impairment cohorts but were not permitted to be matched to more than 1 subject within the same hepatic impairment group.

### 2.2. Inclusion and Exclusion Criteria

Subjects included males or females of non-childbearing potential, between 18 and 74 years of age (inclusive), in good health, except for additional or specific inclusion criteria related to hepatic impairment subjects, as determined by the investigator based on no clinically significant findings from the medical history, physical examination, and 12-lead ECG.

Key exclusion criteria included a history of stomach or intestinal surgery or resection that would have potentially altered the absorption and/or excretion of orally administered drugs; history or presence of an abnormal ECG; use of moderate/strong CYP3A4 inhibitors or inducers and all P-gp inhibitors within 5 half-lives or 14 days, whichever was longer, prior to Check-in (Day −1); history of alcoholism or drug addiction within 1 year prior to Check-in (Day −1); QTcF > 480 ms demonstrated on at least two ECGs that was clinically significant by the investigator’s opinion. Subjects abstained from consuming alcohol- or caffeine-containing foods and beverages for 72 h prior to Check-in. Subjects did not receive any investigational study drug within 5 half-lives or 30 days, whichever was longer, prior to Check-in until Study Completion. For subjects with hepatic impairment, additional exclusion criteria included evidence of progressive liver disease; requirement for additional medication for hepatic encephalopathy; or total bilirubin levels > 6 mg/dL.

### 2.3. Safety and Tolerability

Safety was assessed by the review of adverse events (AEs), vital signs, clinical laboratory assessments, ECGs, and physical examinations. A treatment-emergent AE (TEAE) was defined as an AE that started during or after dosing, or started prior to dosing and increased in severity after dosing. A treatment-related TEAE was a TEAE related to the study treatment as determined by the investigator. All AEs were assigned a severity grade using the National Cancer Institute Common Terminology Criteria for Adverse Events Version 5.0. The frequency of subjects with TEAEs were summarized by hepatic function cohort. Descriptive statistics were calculated for the safety parameters. No formal statistical analyses were planned or performed for the safety data since the study was not powered.

### 2.4. Analytical Methods

#### 2.4.1. Plasma PK

The concentrations of pralsetinib in human plasma were measured using a validated liquid chromatography–tandem mass spectrometry (LC-MS/MS) assay. Pralsetinib and its internal standard ([^13^C]-Pralsetinib-d_3_) were extracted from human plasma by supported-liquid extraction (SLE). The concentrations were calculated with the use of a standard curve with a 1/x2 linear regression over a concentration range of 2 to 2000 ng/mL. The mass spectrometer was operated in positive electrospray ionization (ESI) mode under the optimized conditions with multiple reaction monitoring (MRM) of the analytes and internal standards. The precision and accuracy of the assays were satisfactory throughout the study. Additional details on this validated assay are presented in Supplementary Material S1.

#### 2.4.2. Plasma Protein Binding

As per the FDA and EMA guidance, clinical plasma samples (3 and 24 h postdose) were assessed for the extent of binding of pralsetinib to plasma proteins using a rapid equilibrium dialysis (RED) device (Thermo Fisher Scientific, Waltham, MA, USA) [9,11]. Briefly, 200 μL plasma sample was added to the donor side and 400 μL DPBS buffer (Corning Life Science, Tewksbury, MA, USA) was added to the receiver side. The dialysis plate was then sealed and incubated at 37 °C, 5% CO_2_, saturated humidity and 300 rpm for 6 h. After that, the plasma and dialysate were collected and the matrix was matched to plasma:DPBS (1:1) prior to analysis. The pralsetinib concentrations in the matched matrix were measured using a different validated LC-MS/MS assay with plasma:DPBS (1:1) as the matrix and a curve range of 0.2 to 200 ng/mL. All protein binding determinations were performed in quadruplicate to ensure accurate results.

### 2.5. Pharmacokinetic Statistical Methodology

PK blood samples were collected before dosing and at 0.5, 1, 2, 3, 4, 6, 8, 12, 24, 36, 48, 71, 96, 144, and 192 h after dosing. PK parameters for total pralsetinib were determined from the plasma concentration using non-compartmental methods in Phoenix WinNonlin (Certara, Version 8.3.5).

The primary analysis was to determine the PK of pralsetinib following the administration of a single oral dose of 200 mg (100 mg × 2 capsules) to subjects with moderate or severe hepatic impairment, as defined by the Child–Pugh classification, compared to demographically matched healthy subjects with normal hepatic function. The primary PK parameters (AUC and C_max_) were log (ln)-transformed [12] and analyzed using an analysis of variance model in accordance with the regulatory guidance [9,11,13]. Regression analysis that evaluated the correlation between the ln-transformed AUC and C_max_ versus baseline Child-Pugh total score was performed; Spearman’s rank correlation coefficient at its *p*-value were calculated. For each PK parameter separately, the least squares mean (LSM) for each hepatic function cohort, difference in LSMs between the test and reference hepatic function cohorts, and corresponding 90% CIs were calculated; these values were then back-transformed to give the geometric LSM, geometric mean ratio (GMR) and corresponding 90% CI. These calculations were performed using SAS version 9.4 (SAS Institute, Cary, NC, USA).

## 3. Results

### 3.1. Study Population

A total of 29 subjects were enrolled in this study, including 9 subjects with moderate hepatic impairment (Child–Pugh Class B), 6 subjects with severe hepatic impairment (Child–Pugh Class C), and 14 matched subjects with normal hepatic function. The demographics were similar across the groups with the exception of race. All of the subjects with moderate or severe hepatic impairment were White, while the majority of subjects with normal hepatic function were White (71.4%) (Table 1). All 29 subjects were dosed and were included in the safety evaluation (“safety evaluable population”). Twenty-eight subjects completed the study despite the fact that one subject with moderate hepatic impairment per Child–Pugh classification was incorrectly dosed. One subject with normal hepatic function discontinued one day early due to withdrawal by the subject. Accordingly, 13 of the 14 subjects with normal hepatic function and eight of the nine subjects with moderate hepatic impairment were included in the PK statistical analysis (“PK evaluable population”).

### 3.2. Pharmacokinetics

**Figure 2:**
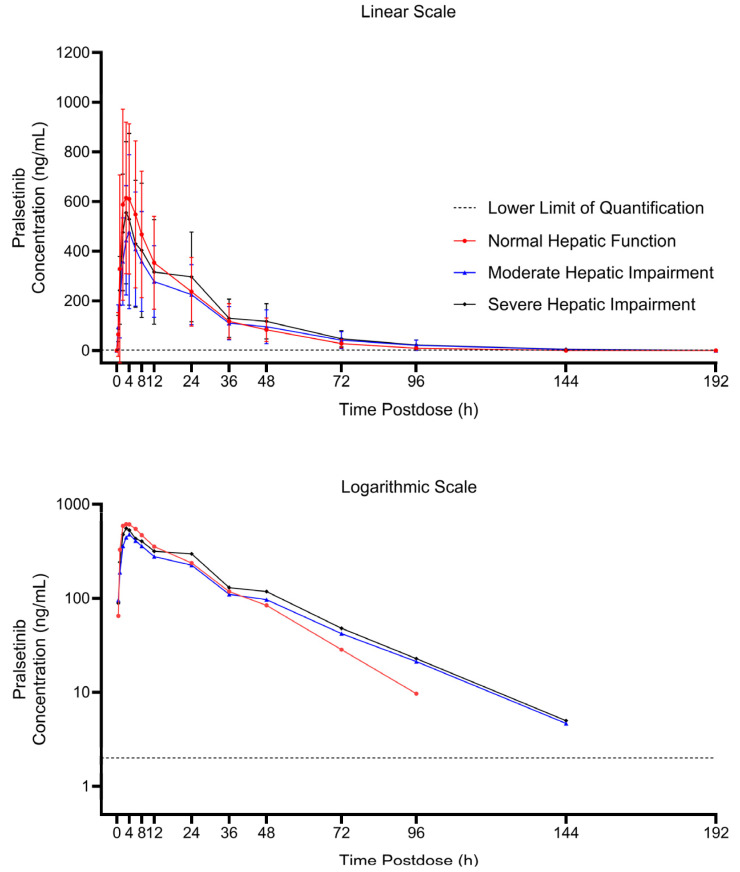
Arithmetic mean (+SD) concentration–time profiles of pralsetinib presented as both linear and logarithmic scales following a 200 mg dose of pralsetinib in subjects with hepatic impairment classified by Child–Pugh Score and subjects with normal hepatic function.

The mean pralsetinib plasma concentration–time profiles up to 192 h after administration of a 200 mg dose of pralsetinib in subjects with hepatic impairment classified by Child–Pugh scores as moderate or severe were very similar to those with normal hepatic function as shown in Figure 2. After reaching C_max_, the plasma concentrations of pralsetinib appeared to decline in a multiphasic manner in all subjects. The geometric mean t_1/2_ was similar in subjects with normal hepatic function and subjects with moderate or severe hepatic impairment, ranging from 15.4 to 18.9 h (Table 2).

Hepatic impairment severity: moderate = Class B (Child–Pugh total score 7–9, inclusive); severe = Class C (Child–Pugh total score 10 to 15, inclusive).

The statistical analysis of the effect of hepatic impairment, classified by Child–Pugh scores, on PK parameters for pralsetinib is presented in Table 3. Compared to subjects with normal hepatic function, C_max_ was similar in subjects with moderate hepatic impairment and lower in subjects with severe hepatic impairment, with geometric mean ratios (GMRs) of 0.986 and 0.679, respectively. AUC_0–∞_ were similar in subjects with moderate hepatic impairment and subjects with severe hepatic impairment compared to subjects with normal hepatic function, with GMRs of 1.12 and 0.858, respectively. In general, as assessed from the geometric coefficient of variation (%CV), between-subject variability was high for C_max_ and AUC_0–∞_ for all subjects in this study, ranging from 56.5% to 72.0%.

**Figure 3:**
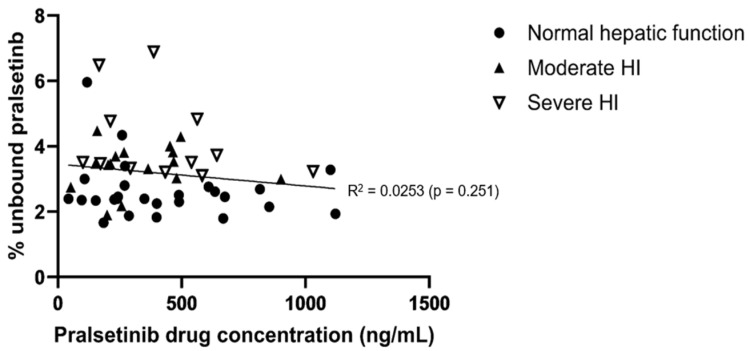
Individual fraction unbound vs. pralsetinib drug concentrations in subjects with normal, moderate or severe hepatic function.

Unbound C_max_ and unbound AUC_0–∞_ and the associated statistical analysis of pralsetinib classified based on Child–Pugh score are presented in Table 2. The geometric mean of the fraction unbound (fu) was approximately 31% or 60% higher for subjects with moderate or severe hepatic impairment, respectively, compared to subjects with normal hepatic function. The higher fu values resulted in numerically higher systemic exposure to unbound pralsetinib in subjects with moderate or severe hepatic impairment compared to subjects with normal hepatic function, based on GMRs ranging from 1.14 to 1.31 for subjects with moderate hepatic impairment and from 1.29 to 1.64 for subjects with severe hepatic impairment. Pralsetinib protein binding was not dependent on the drug concentration over a range of total pralsetinib plasma concentration from 41.9 to 1120 ng/mL (Figure 3).

Regression analysis using Child–Pugh scores was performed and it suggested no evidence of a relationship between bound and unbound C_max_ or AUC_0–∞_ and Child–Pugh total score (all *p*-values > 0.3, Figure S1).

### 3.3. Analysis Based on NCI-ODWG Criteria

Exploratory analysis was conducted where subjects were re-classified according to the NCI-ODWG for Hepatic Dysfunction criteria. After reclassification based on the NCI-ODWG criteria, all subjects who were initially categorized as having normal hepatic function and severe hepatic impairment based on the Child–Pugh classification remained in their respective cohorts. Consequently, the PK profiles, PK parameters, and exploratory statistical analysis for these cohorts were the same as the primary analysis using the Child–Pugh scores. Three subjects from the moderate hepatic impairment cohort per the Child–Pugh classification were reclassified to mild hepatic impairment based on the NCI-ODWG criteria.

Subjects with moderate hepatic impairment (n = 5) had similar C_max_ and AUC_0–∞_ compared to subjects with normal hepatic function. The GMRs for C_max_ (1.00; 90% CI 0.600 to 1.68) and AUC_0–∞_ (1.22; 90% CI 0.742 to 2.01) in this exploratory statistical analysis (based on NCI-ODWG) were similar to that in the primary statistical analysis (based on Child–Pugh). For the three subjects who had been reclassified to mild HI based on NCI-ODWG, their GMRs (90% CI) for C_max_ and AUC_0–∞_ when compared to their matched subjects with normal hepatic function were 0.904 (0.445, 1.83) and 1.02 (0.441, 2.35), respectively.

The fu of pralsetinib was approximately 35%, 29%, and 60% higher for subjects with mild, moderate, and severe hepatic impairment, respectively, by the NCI-ODWG criteria compared to subjects with normal hepatic function. Pralsetinib exposures were similar between subjects with moderate hepatic impairment by the NCI-ODWG criteria and subjects with normal hepatic function, with GMRs of 1.15 (90% CI 0.678 to 1.93) for C_max,u_ and 1.39 (90% CI 0.851 to 2.28) for AUC_0–∞,u_.

Regression analysis suggested that there was no significant relationship between the total and unbound C_max_ or AUC_0–∞_ and the NCI-ODWG classification (data on file; all *p*-values > 0.2).

### 3.4. Safety and Tolerability

Overall, a single oral dose of 200 mg pralsetinib was safe and well tolerated in subjects with normal hepatic function and in those with moderate or severe hepatic impairment. There were no serious AEs, and no subjects withdrew due to an AE. Overall, seven TEAEs were reported in six subjects (20.7%) with one TEAE in one (11.1%) subject with moderate hepatic impairment, three TEAEs in two subjects (33.3%) with severe hepatic impairment, and three TEAEs in three subjects (21.4%) with normal hepatic function. The majority of the TEAEs were mild (Severity Grade 1) or moderate (Severity Grade 2) in intensity. There was one Severity Grade 3 TEAE of hepatic encephalopathy in a subject with severe hepatic impairment that the investigator judged to be not related to the study drug. All TEAEs resolved by the end of the study.

## 4. Discussion

Pralsetinib is mainly eliminated via the hepatobiliary pathway with approximately 73% of the total administered radioactive dose recovered in the feces. Population pharmacokinetic analysis suggested that mild hepatic impairment did not impact the pralsetinib PK. In this dedicated HI study, the PK of pralsetinib in subjects with moderate or severe hepatic impairment was compared with subjects with normal hepatic function following the administration of a single oral dose of 200 mg pralsetinib to provide dosing recommendations for pralsetinib in patients with hepatic impairment.

The recommended pralsetinib dose for the treatment of patients with metastatic RET fusion-positive NSCLC or thyroid cancer is 400 mg orally once daily [2,3]; however, a lower dose of 200 mg pralsetinib was administered to subjects enrolled in this study. In previous studies, single doses of pralsetinib of 200 mg, 300 mg and 400 mg have been given to healthy subjects, with all three doses shown to be safe and well tolerated [3]. Pralsetinib has been shown to demonstrate dose proportionality across the range of 200 to 400 mg [3]. Given that an increase in exposure was expected in subjects with hepatic impairment and to limit unnecessarily high drug exposure in non-cancer subjects, the lowest dose within the linearity range was selected. Based on a pooled popPK analysis that included data from patients with RET-altered thyroid cancers, patients with RET-fusion NSCLC and healthy subjects in different pralsetinib studies, disease status does not have a clinically relevant impact on the PK of pralsetinib [3]. Hence, the PK results from this study can be extrapolated from the evaluated dose of 200 mg to the approved dose of 400 mg, as well as from healthy subjects to patients indicated for treatment with pralsetinib.

Following a single oral dose of 200 mg pralsetinib, the drug was rapidly absorbed with a T_max_ of approximately 3 h post dose in all cohorts, and the plasma concentrations appeared to then decline in a multiphasic manner in all subjects. Given the small sample size and the high inter-subject variability of pralsetinib, caution should be used when interpreting this observation. In fact, based on the population PK model for pralsetinib, which included 491 subjects (193 healthy subjects, 161 patients with NSCLC, 124 patients with RET-mutation positive MTC and 13 patients with RET-fusion positive thyroid) with 7566 quantifiable pralsetinib concentrations pooled from 5 clinical studies, the PK of pralsetinib was characterized by a one-compartment model with linear elimination [3,14].

Compared to subjects with normal hepatic function, the C_max_ and AUC were similar in subjects with moderate hepatic impairment. While the AUC of pralsetinib was also similar in subjects with severe hepatic impairment compared to those with normal hepatic function, the C_max_ was lower in subjects with severe hepatic impairment, with a GMR of 0.679. This difference could be difficult to interpret due to the observed high inter-individual variability in the pralsetinib PK across all cohorts. Lower C_max_ in patients with hepatic impairment has been observed in other studies previously [15,16]. Potential changes in the intestinal permeability that are related to conditions that patients with hepatic impairment may be at increased risk for and changes in the activity of metabolic enzymes or transporters (such as P-gp, of which pralsetinib is a substrate) in the intestine and liver, in patients with hepatic impairment may limit drug absorption, resulting in a lower C_max_ in these subjects [17,18].

Discordance between the Child–Pugh classification and NCI-ODWG criteria have been reported previously, with the latter having more subjects being classified as less hepatically impaired compared to the Child–Pugh classification system [19]. The Child–Pugh classification is based on two subjective clinical features (encephalopathy and ascites) and three biochemical measures (serum bilirubin, serum albumin, and INR) whereas NCI-ODWG is based on only two biochemical markers (total bilirubin and AST). In this study, subjects with hepatic impairment were enrolled and the data were analyzed based on the Child–Pugh classification. Then, an exploratory analysis was performed by re-stratifying subjects using the NCI-ODWG criteria. In this study, three of the eight subjects with moderate HI per the Child–Pugh classification were reclassified as mild HI under the NCI-ODWG criteria. Despite such differences in the number of subjects in the moderate HI cohort in the exploratory analysis, the overall impact of moderate HI on the exposure to pralsetinib was consistent between the two classification systems. Further, the changes in systemic exposure in the three subjects with mild HI classified with the NCI-ODWG criteria were also not significant, which is consistent with previous findings from the popPK analysis.

Regulatory guidance suggests that subjects in the control group (i.e., normal liver function) be matched to the HI subjects; however, there are no recommendations on how PK should be compared between the HI and control groups. While there are published studies that calculated AUC and C_max_ GMR using all enrolled subjects with normal liver function as the reference group, there are also a few, including this study, that only included subjects who were matched to a subject within a specific HI test group [20,21]. Specifically for this study, given the small sample size and known significant covariate effects of age, body weight, and sex, a comparison with matched normal subjects was deemed more appropriate.

In accordance with regulatory guidance, the fu of pralsetinib at the trough concentration and maximal concentration were measured [9]. Pralsetinib protein binding was not dependent on the total pralsetinib concentration over the range of values assessed. The fu of pralsetinib was approximately 31% or 60% higher for subjects with moderate or severe hepatic impairment, respectively, compared to subjects with normal hepatic function. These changes in the fu of pralsetinib are in line with the differences in the baseline albumin levels in the moderate and severe hepatic impairment cohorts compared to the subjects with normal hepatic function (Table 1). The higher fu values resulted in numerically higher systemic exposure to unbound pralsetinib (Table 3) and consequently faster total drug clearance. This further explained the observed marginal decrease in the total pralsetinib exposure in subjects with severe hepatic impairment in this study.

In addition, while the unbound pralsetinib exposure in subjects with severe hepatic impairment was higher than in those with normal liver function, it overlapped with the range of estimated unbound exposure observed in other pralsetinib studies after adjusting for dose and drug accumulation due to multiple dosing (data on file). The 90% confidence intervals for the geometric mean ratios comparing the pralsetinib exposure in subjects with moderate or severe hepatic impairment to those with normal liver function, albeit wide, spanned unity. Hence, similarity in systemic exposure between subjects with severe hepatic impairment and subjects with normal hepatic function could not be ruled out. However, between-subject variability was high across the three cohorts. In addition, no safety concerns were observed in this study. While this study was not powered to evaluate the safety and tolerability of pralsetinib in subjects with hepatic impairment and those with normal liver function, there was no obvious pattern of TEAEs within or across the cohorts. When interpreting these results, it needs to be considered that this was a small single-dose study conducted in subjects of generally good health except for their hepatic function. However, the TEAEs that were observed were in line with the known safety profile of pralsetinib or the underlying hepatic disease of the subjects. Overall, given the lack of meaningful change in the total exposure, high inter-subject variability, and consistent safety profile, the modest change in the unbound pralsetinib exposure in subjects with severe hepatic impairment is determined to be not clinically relevant, thus not warranting a dose adjustment in these patients.

## Figures and Tables

**Table 1:** Summary of Characteristics of Subjects Enrolled in the Hepatic Impairment Study.

Demographic	Normal Hepatic Function		Moderate Hepatic Impairment		Severe Hepatic Impairment (N = 6)
	Safety Evaluable Population (N = 14)	PK Evaluable Population (N = 13) *	Safety-Evaluable Population (N = 9)	PK-Evaluable Population (N = 8) *	
Age (years)	56.9 (9.31)	57.2 (9.65)	57.4 (11.76)	56.4 (12.09)	60.8 (6.85)
Sex					
Male	10 (71.4%)	10 (76.9%)	7 (77.8%)	6 (75%)	5 (83.3%)
Female	4 (28.6%)	3 (23.1%)	2 (22.2%)	2 (25%)	1 (16.7%)
Race					
White	10 (71.4%)	9 (69.2%)	9 (100%)	8 (100%)	6 (100%)
Black or African American	4 (28.6%)	4 (30.8%)	---	---	---
Ethnicity					
Hispanic or Latino	7 (50.0%)	7 (53.8%)	5 (55.6%)	5 (62.5%)	3 (50.0%)
Not Hispanic or Latino	7 (50.0%)	6 (46.2%)	4 (44.4%)	3 (37.5%)	3 (50.0%)
Height (cm)	172.15 (9.809)	171.93 (10.174)	169.96 (7.434)	169.53 (7.321)	172.18 (11.519)
Body Weight (kg)	86.26 (15.288)	85.92 (15.850)	90.52 (17.360)	88.5 (16.266)	88.17 (21.017)
Body Mass Index (kg/m^2^)	29.035 (3.9794)	28.777 (3.9064)	31.272 (4.9841)	30.750 (4.7247)	29.372 (3.9129)
Baseline Albumin (g/L)	42.9 (3.17)	43.2 (3.18)	35.1 (3.69)	35.0 (3.54)	29.5 (4.59)

N = number of subjects. Body mass index (kg/m^2^) = body weight (kg)/height (m)^2^. For continuous data, mean (SD) statistics are presented; for categorical data, n (%) statistics are presented. * One subject with normal hepatic function, who met the exclusion criterion for previous participation in another investigational study drug trial, and 1 subject with moderate hepatic impairment, who was dosed with 100 mg of pralsetinib instead of 200 mg, were excluded from the PK descriptive statistics and PK statistical analysis. Accordingly, 13 of the 14 subjects with normal hepatic function and eight of the nine subjects with moderate hepatic impairment were included in the PK statistical analysis.

**Table 2:** Summary of Pharmacokinetic Parameters of Pralsetinib—Child–Pugh Score.

Parameter	Normal Hepatic Function	Moderate Hepatic Impairment	Severe Hepatic Impairment
N	13	8	6
AUC_0–∞_ (h·ng/mL)	12,400 (61.6)	11,400 (72.0)	13,700 (62.1)
C_max_ (ng/mL)	619 (67.3)	476 (56.5)	508 (70.1)
T_max_ (h)	3.00 (1.00–6.07)	3.00 (1.00–6.00)	3.50 (3.00–4.00)
t_1/2_ (h)	15.4 (25.1)	17.5 (41.1)	18.9 (26.6)
fu	0.0253 (27.5)	0.0331 (23.1)	0.0404 (27.3)
AUC_0–∞,u_ (h·ng/mL)	314 (53.8)	379 (77.6)	552 (55.4)
C_max,u_ (ng/mL)	15.6 (52.7)	15.7 (63.3)	20.5 (69.2)
CL/F (L/h)	16.1 (61.6)	17.5 (72.0)	14.6 (62.1)
Vz/F (L)	358 (55.6)	443 (65.1)	398 (51.8)

AUC_0–∞_ = area under the concentration–time curve from time 0 extrapolated to infinity; AUC_0–∞,u_ = area under the concentration–time curve from time 0 extrapolated to infinity of free drug; CL/F = apparent systemic clearance; C_max_ = maximum observed concentration; C_max,u_ = maximum observed concentration of free drug; fu = fraction of unbound drug; t_1/2_ = apparent terminal elimination half-life; t_max_ = time to maximum observed concentration; Vz/F = apparent volume of distribution during the terminal elimination phase; geometric mean (CV) [n] statistics presented; for t_max_, median (minimum–maximum) [n] statistics presented. Hepatic impairment severity: moderate = Class B (Child–Pugh total score 7 to 9, inclusive); severe = Class C (Child–Pugh total score 10 to 15, inclusive).

**Table 3:** Statistical Analysis of Pharmacokinetic Parameters of Pralsetinib—Child–Pugh Score.

Parameter	Comparison	Hepatic Function Cohort	n	GLSM (CV)	Test Versus Reference GMR (90% CI)
AUC_0–∞_ (h·ng/mL)	Moderate vs. Normal	Normal Hepatic Function (Reference)	8	10,200 (63.3)	1.12 (0.654, 1.93)
		Moderate Hepatic Impairment (Test)	8	11,400 (72.0)	
	Severe vs. Normal	Normal Hepatic Function (Reference)	6	15,900 (42.1)	0.858 (0.511, 1.44)
		Severe Hepatic Impairment (Test)	6	13,700 (62.1)	
C_max_ (ng/mL)	Moderate vs. Normal	Normal Hepatic Function (Reference)	8	482 (67.1)	0.986 (0.597, 1.63)
		Moderate Hepatic Impairment (Test)	8	476 (56.5)	
	Severe vs. Normal	Normal Hepatic Function (Reference)	6	748 (68.1)	0.679 (0.353, 1.31)
		Severe Hepatic Impairment (Test)	6	508 (70.1)	
AUC_0–∞,u_ (h·ng/mL)	Moderate vs. Normal	Normal Hepatic Function (Reference)	8	290 (58.4)	1.30 (0.756, 2.25)
		Moderate Hepatic Impairment (Test)	8	379 (77.6)	
	Severe vs. Normal	Normal Hepatic Function (Reference)	6	339 (41.6)	1.63 (0.987, 2.68)
		Severe Hepatic Impairment (Test)	6	552 (55.4)	
C_max,u_ (ng/mL)	Moderate vs. Normal	Normal Hepatic Function (Reference)	8	13.8 (61.7)	1.14 (0.690, 1.90)
		Moderate Hepatic Impairment (Test)	8	15.7 (63.3)	
	Severe vs. Normal	Normal Hepatic Function (Reference)	6	15.9 (56.2)	1.29 (0.704, 2.36)
		Severe Hepatic Impairment (Test)	6	20.5 (69.2)	

AUC_0–∞_ = area under the concentration on-time curve from time 0 extrapolated to infinity; AUC_0–∞,u_ = area under the concentration on-time curve from time 0 extrapolated to infinity of free drug; CI = confidence interval; C_max_ = maximum observed concentration; C_max,u_ = maximum observed concentration of free drug; CV= geometric coefficient of variation; GLSM = geometric least square mean; GMR = geometric mean ratio; n = number of subjects with valid observations. The comparison reference group (i.e., normal hepatic function group) only included those subjects who were matched to a subject within each specific test group. The GLSMs, GMRs, and corresponding CIs were obtained by taking the exponential of the least square means (LSMs) differences in LSMs and corresponding CIs on the natural log (ln) scale.

## Data Availability

Qualified researchers may request access to individual patient level data through the clinical study data request platform (https://vivli.org/). Further details on Roche’s criteria for eligible studies are available here (https://vivli.org/members/ourmembers/). For further details on Roche’s Global Policy on the Sharing of Clinical Information and how to request access to related clinical study documents, see here (https://www.roche.com/research_and_development/who_we_are_how_we_work/clinical_trials/our_commitment_to_data_sharing.html).

## Supplementary Materials

The following supporting information can be downloaded at: https://www.mdpi.com/article/10.3390/pharmaceutics16040564/s1, Supplementary Material S1: Details on validated liquid chromatography-tandem mass spectrometry assay to measure the concentration of pralsetinib in human plasma. Figure S1: Regression analysis of pralsetinib pharmacokinetic parameters. Scatterplots of total AUC_0–∞_ (A), Cmax (B), unbound AUC_0–∞_ (C), unbound Cmax (D) versus screening Child–Pugh total score are presented. There was no evidence of a relationship between total and unbound AUC and Cmax. The Spearman’s ranks coefficient indicated very weak correlations between total and unbound AUC or Cmax and Child–Pugh total score. Additionally, the *p*-values for the slopes ranged from 0.35 to 0.90.
