# Supplementary material for: Exploring the Impact of Hepatic Impairment on Pralsetinib Pharmacokinetics
Source: Pharmaceutics. 2024 Apr 20;16(4):564. doi: 10.3390/pharmaceutics16040564 (PMC11053887; doi:10.3390/pharmaceutics16040564)
Supplement: Supplementary file 1 [file pharmaceutics-16-00564-s001.zip › pharmaceutics-2939167-supplementary.pdf]

Supplemental Materials

Supplementary Material S1: Details on validated liquid chromatography-tandem mass spectrometry assay to measure the concentration of pralsetinib in human plasma.

- Curve range: 2.00 (LLOQ) to 2000 (ULOQ) ng/mL.
- Inter-Assay Accuracy (%Bias): 1.3% to 5.0%
- Inter-Assay Precision (%RSD): 4.5% to 8.9%
- Matrix effect:

Nominal con. of 2 ng/mL, 6 individual blank matrix spiked at the LLOQ level						
Result (ng/mL)	1.84	2.06	1.97	1.66	1.88	1.94
Bias (%)	-8	3	-1.5	-17	-6	-3

- Recovery: LQC: 84.9%,
  LMQC: 83.3%,
  MQC: 90.5%,
  HQC: 84.1%

- Matrix bench-top stability: 24 hours at room temperature
- Matrix free-thaw stability: 5 cycles at -10°C to -30°C, 5 cycles at -60°C to -80°C.
- Matrix frozen stability: 750 days at -10°C to -30°C, 750 days at -60°C to -80°C.

- Representative chromatograms:

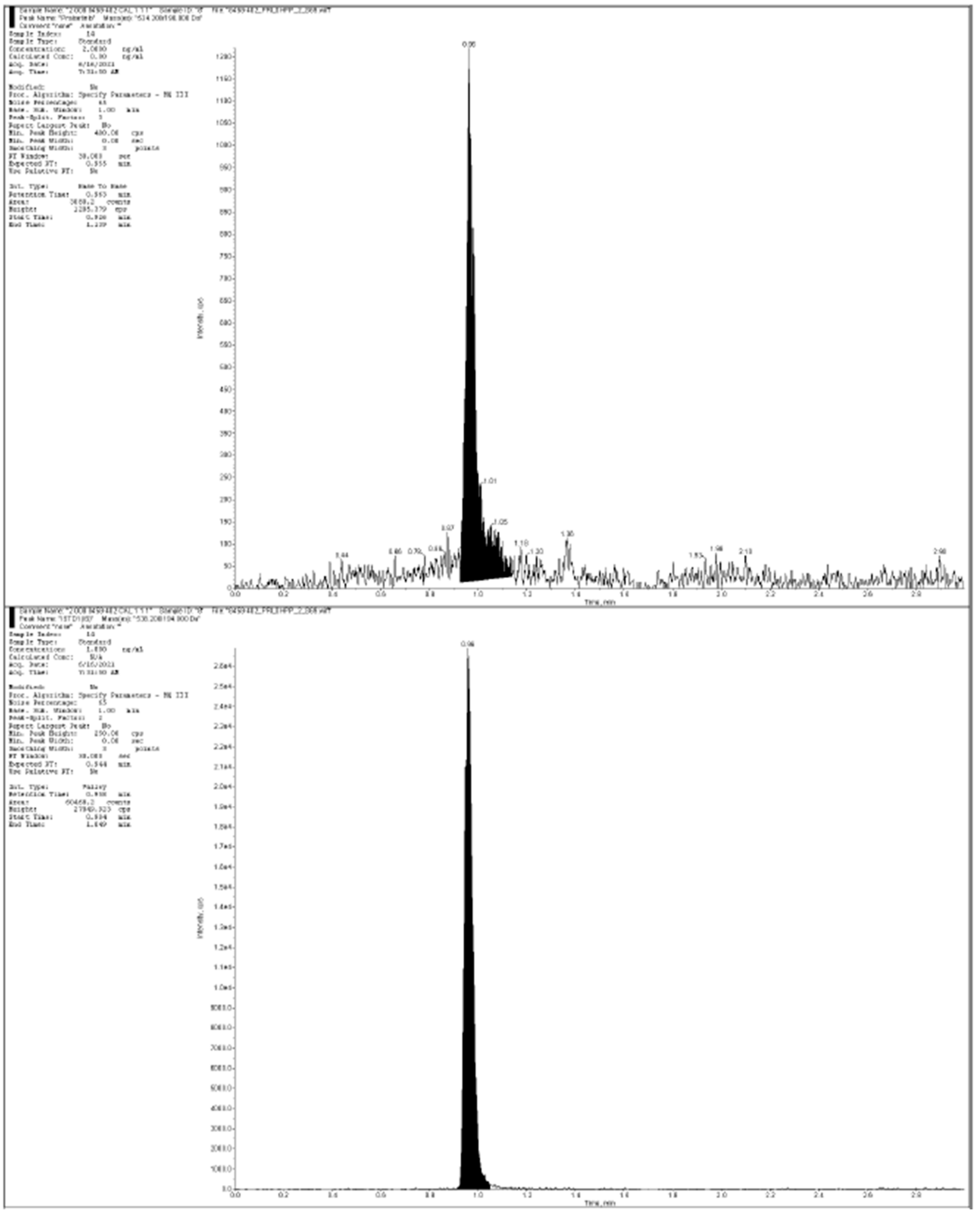

Chromatogram of a 2.00 ng/mL Calibration Standard (Lower Limit of Quantification)

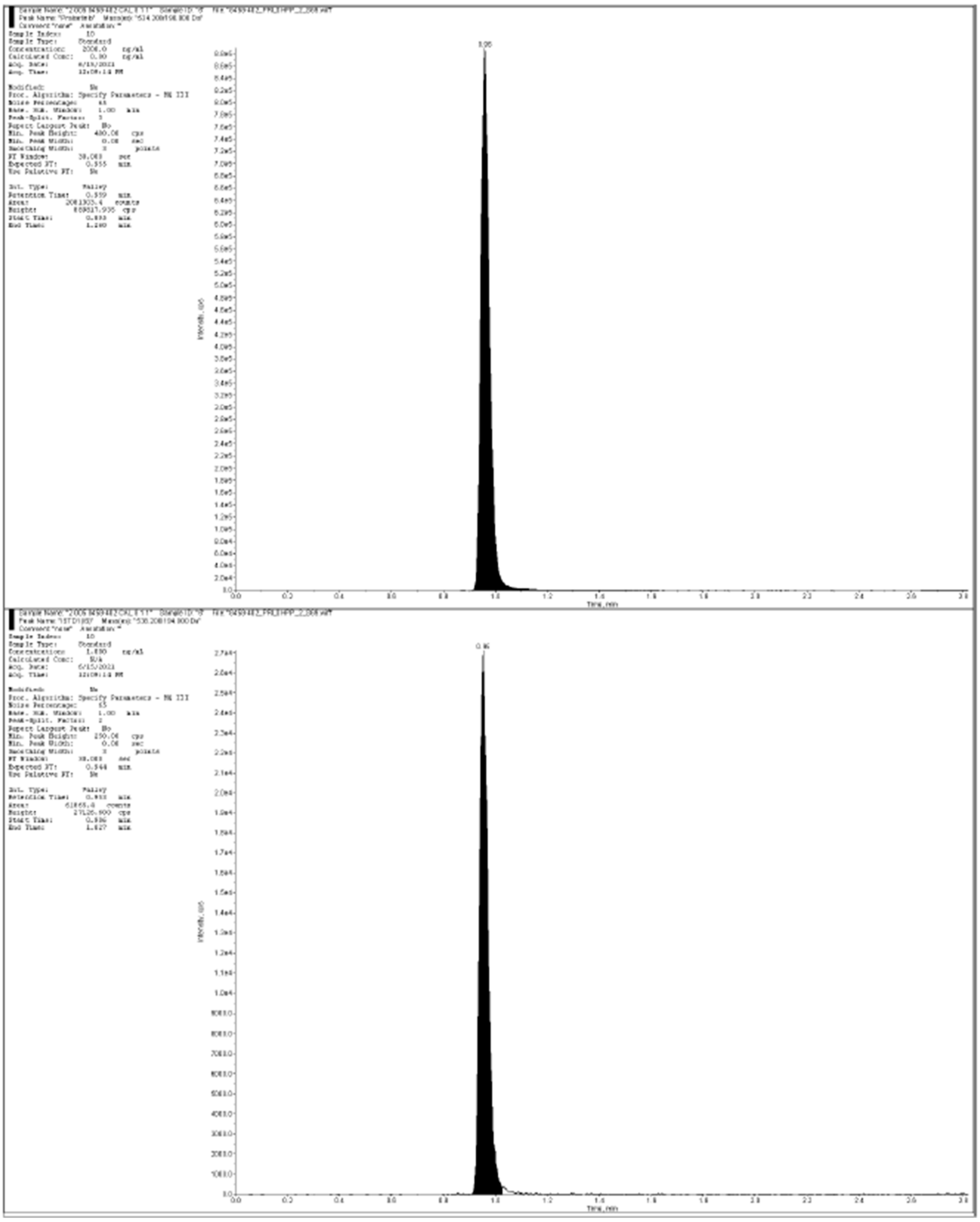

Chromatogram of a 2000 ng/mL Calibration Standard (Upper Limit of Quantification)

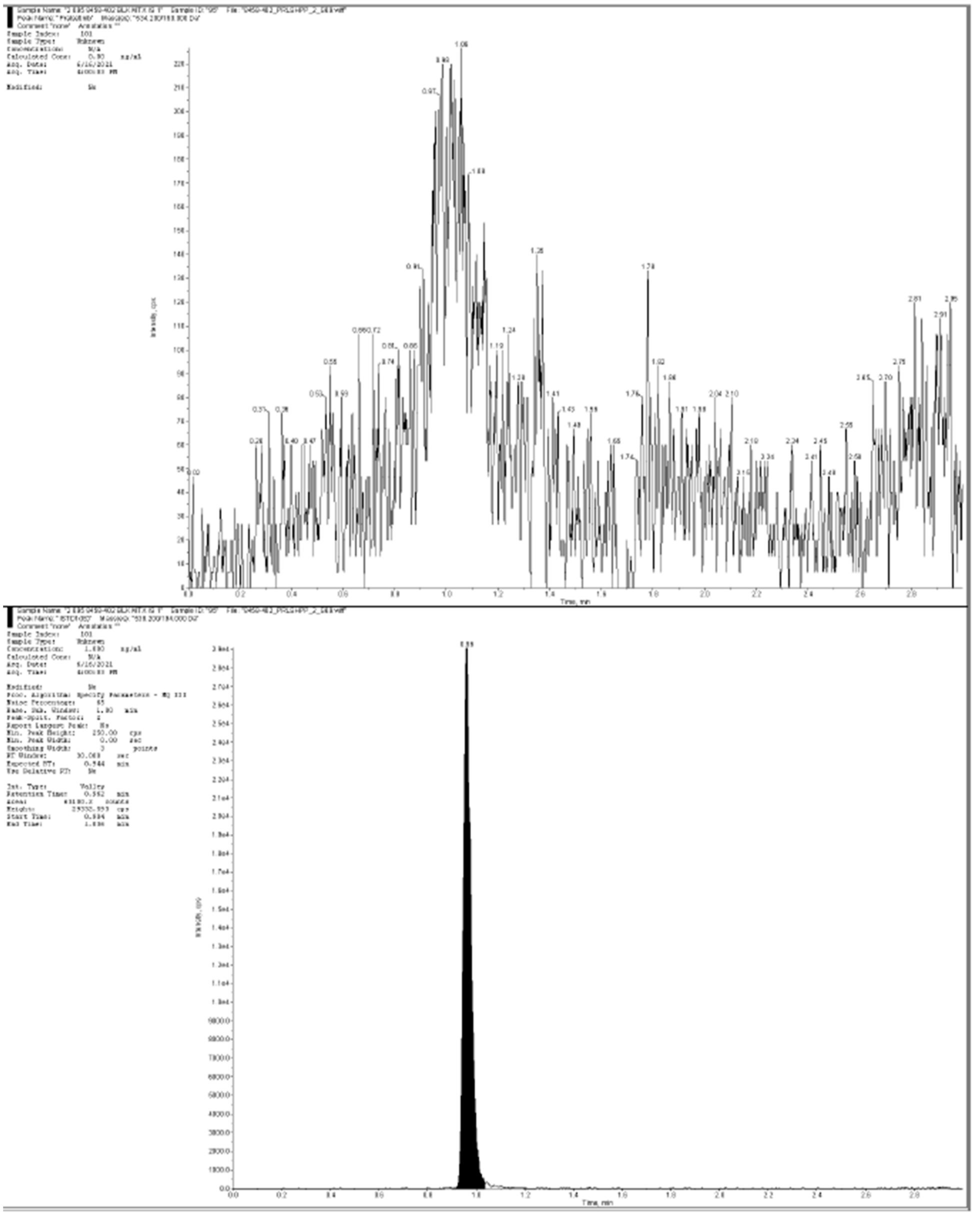

Chromatogram of a Control Zero Blank

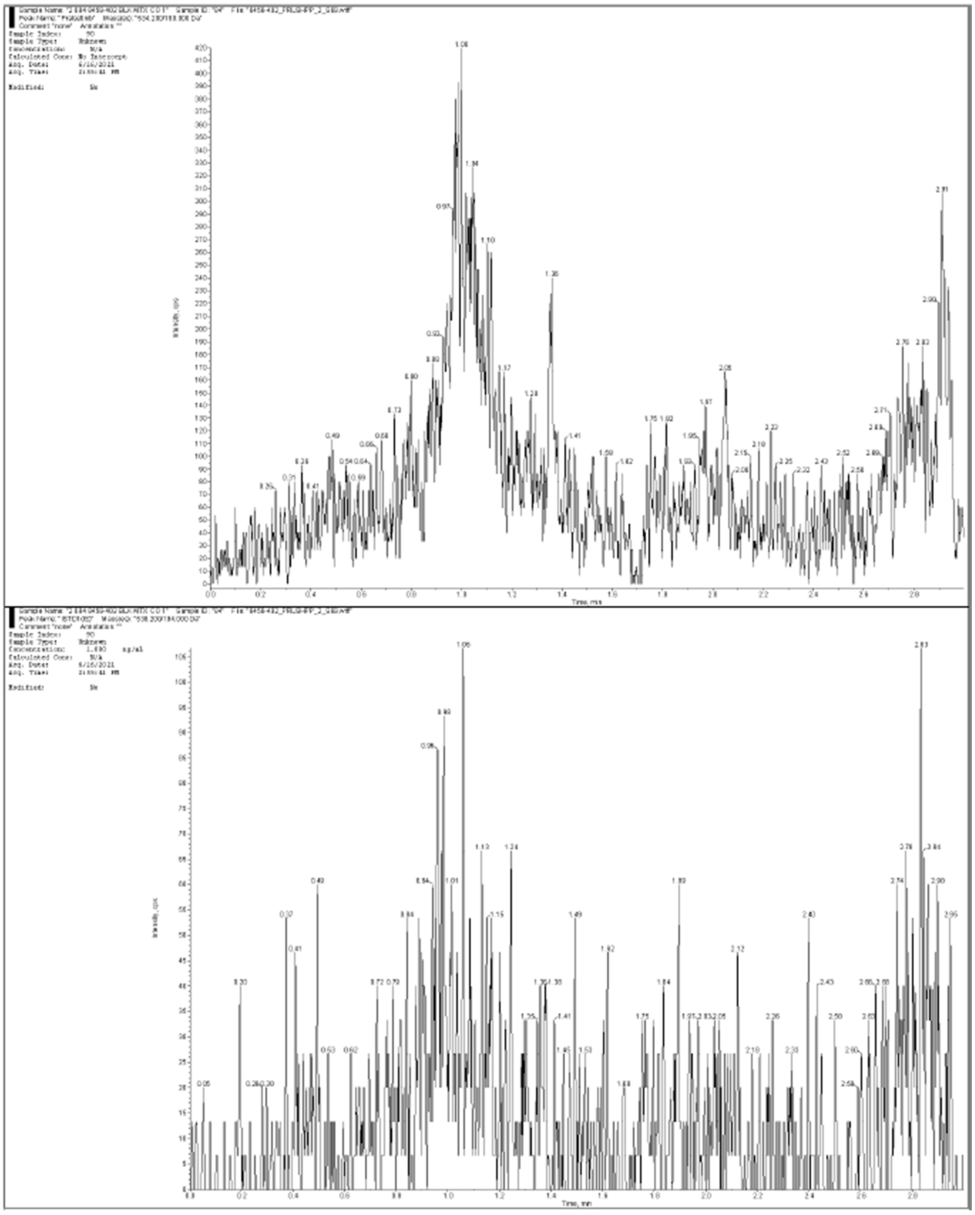

Chromatogram of a Carryover Blank Injected Immediately Following the Highest Calibration Standard

Figure S1. Regression analysis of pralsetinib pharmacokinetic parameters. Scatterplots of total $AUC_{0-\infty}$ (A), C_{max} (B), unbound $AUC_{0-\infty}$ (C), unbound C_{max} (D) versus screening Child-Pugh total score are presented. There was no evidence of a relationship between total and unbound AUC and C_{max}. The Spearman's ranks coefficient indicated very weak correlations between total and unbound AUC or C_{max} and Child-Pugh total score. Additionally, the p-values for the slopes ranged from 0.35 to 0.90.

A

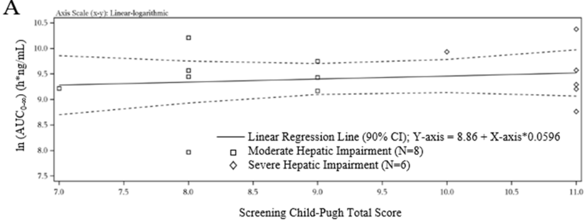

P-value (90% CI) for the linear regression slope: 0.6232 (-0.151, 0.270)
Spearman's rank coefficient: 0.059

B

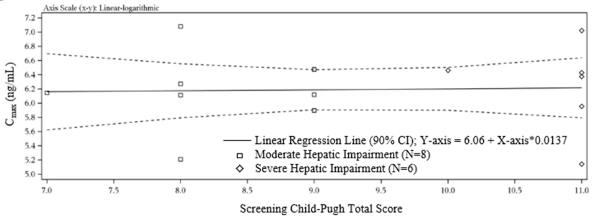

P-value (90% CI) for the linear regression slope: 0.9027 (-0.182, 0.210)
Spearman's rank coefficient: 0.050

C

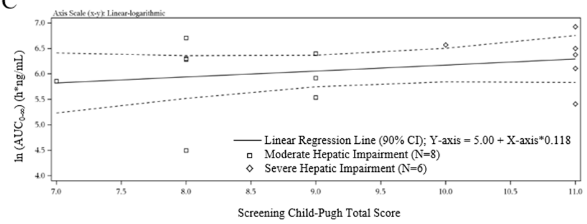

P-value (90% CI) for the linear regression slope: 0.3479 (-0.0971, 0.333)
Spearman's rank coefficient: 0.256

D

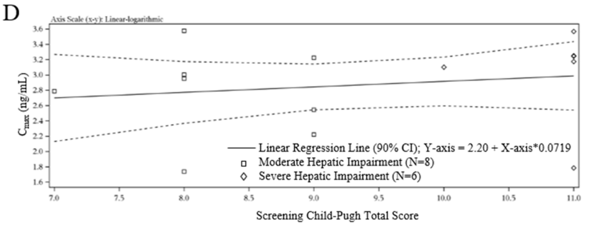

P-value (90% CI) for the linear regression slope: 0.5480 (-0.135, 0.279)
Spearman's rank coefficient: 0.322
